# Experimental drought consistently underestimates productivity responses to natural drought in four Central US grasslands

**DOI:** 10.1007/s00442-025-05746-9

**Published:** 2025-06-19

**Authors:** Kathleen V. Condon, Charles J. W. Carroll, Robert J. Griffin‑Nolan, Ingrid J. Slette, Kate D. Wilkins, Melinda D. Smith, Alan K. Knapp

**Affiliations:** 1 Department of Biology and Graduate Degree Program in Ecology, Colorado State University, Fort Collins, CO USA; 2 Department of Forest and Rangeland Stewardship, Colorado State University, Fort Collins, CO USA; 3 Department of Biological Sciences, California State University, Chico, Chico, CA USA; 4 Department of Ecology, Evolution, and Behavior, University of Minnesota, St. Paul, MN USA; 5 Denver Zoo Conservation Alliance, Denver, CO USA

**Keywords:** Climate change, Drought sensitivity, Grasslands, ANPP, Precipitation, Ecosystem functioning

## Abstract

Climate change is increasing the frequency and severity of droughts globally, and grasslands are particularly vulnerable to such hydrological extremes. Drought effects at the ecosystem scale have been assessed both experimentally and through the study of naturally occurring drought, with emerging evidence that the magnitude of drought effects may vary depending on the approach used. We took advantage of a decadal study of four grasslands to directly contrast responses of aboveground net primary productivity (ANPP) to simulated vs. natural drought. The grasslands spanned a ~ threefold mean annual precipitation gradient (335–857 mm) and were all subjected to a natural 1-year drought (~ 40% reduction in precipitation from the long-term mean) and a 4 year experimental drought (~ 50% precipitation reduction). We expected that the 4 year drought would reduce ANPP more, and that post-drought recovery would be delayed, compared to the 1-year drought. We found instead that the short-term natural drought reduced ANPP more strongly than the simulated drought in all grasslands (~ 10 to ~ 50%) likely due to the co-occurrence of higher temperatures and vapor pressure deficits with reduced precipitation. Post-drought recovery was site specific and each site differed in their recovery from the natural and experimental droughts. These results align with past analyses that experiments that only manipulate soil moisture likely underestimate the magnitude of natural drought events. However, experiments can provide valuable insight into the relative sensitivity of ecosystems to reduced precipitation and soil moisture, a key aspect of drought.

**Supplementary Information:**

The online version contains supplementary material available at 10.1007/s00442-025-05746-9.

## Introduction

As forecasts for more frequent and severe droughts become reality, quantifying the impacts of drought on ecosystems, including patterns of relative drought sensitivity among ecosystems, has become increasingly urgent (Trenberth et al. 2003, 2014; Ault 2020; Chiang et al. 2021; Vicente-Serrano et al. 2022). Ecosystem drought, defined as an abnormal period of low water availability of sufficient duration to potentially alter ecosystem structure and function (Knapp et al. 2024), has been studied in many ways. Approaches include on-the-ground and remote sensing assessments of naturally occurring droughts across a wide range of ecosystem types (e.g., Wolf et al. 2016; Slette et al. 2019; Lu et al. 2021; Hammond et al. 2022) as well as hundreds of smaller-scale manipulative experiments conducted mostly in short-statured grasslands and shrublands (Beier et al. 2012; Knapp et al. 2024; Smith et al. 2024). Although it has long been recognized that different approaches to studying ecological phenomena can lead to variable outcomes (*e.g.,* Sutherland 2006; Fraser et al. 2013), such variation in the assessment of precipitation anomalies has led to persistent concerns about uncertainty in estimates of drought impacts on terrestrial net primary productivity (NPP; *e.g.,* Kröel-Dulay et al. 2022; Smith et al. 2024). Thus, a research priority for ecologists is to increase our understanding of how drought (natural vs. simulated experimentally) impacts NPP, especially aboveground NPP (ANPP), which provides many essential ecosystem services (food, fuel, and fiber) and plays a central role in the global carbon cycle (Zheng et al. 2003; Fahey and Knapp 2007; Knapp et al. 2014; Ahlström et al. 2015).

Small-scale field experiments that reduce precipitation inputs to simulate drought are numerous, but also frequently criticized for a range of shortcomings (Sandel and Smith 2009; Leuzinger et al. 2011; Beier et al. 2012; Hoover et al. 2018; Yang et al. 2022; Kröel-Dulay et al. 2022). Chief among these are that, while the most common infrastructure used to simulate drought (precipitation reduction shelters, Yahdjian and Sala 2006) is effective at lowering soil moisture, this infrastructure has minimal impacts on air temperature, vapor pressure deficit (VPD), and solar radiation (Gherardi and Sala 2013; Alba et al. 2017; Loik et al. 2019) – all of which are important environmental alterations that co-occur during natural drought periods (De Boeck and Verbeeck 2011; Novick et al. 2016; Kröel-Dulay et al. 2022; Wright and Collins 2024). Indeed, high temperatures and VPD can negatively impact plant growth independent of soil water deficits (Grossiord et al. 2020; Schönbeck et al. 2022; Novick et al. 2024) and when combined with extreme reductions in precipitation, “hot droughts” may lead to catastrophic losses in ecosystem productivity (e.g., Breshears et al. 2005; Dannenberg et al. 2022). While experimental approaches offer many advantages and much can be learned from imposing soil moisture deficits to study drought impacts (Knapp et al. 2024), direct comparisons of how ANPP responds to natural vs. experimental (*i.e.*, simulated) droughts are rare. Such comparisons are needed, however, to provide insight into what can and cannot be inferred from these different approaches (*e.g.,* Sandel et al. 2010; Knapp et al. 2018).

Here we conduct such a comparison by taking advantage of a 10-year study (from 2012 to 2021) of four Central US grasslands that span a ~ threefold precipitation gradient. During this period, each of these sites experienced a naturally occurring 1-year extreme drought (~ 40% reduction in precipitation due to a pan-continental drought; Cook et al. 2014; Knapp et al. 2015). Later, all sites were subjected to a simulated 4-year drought (66% reduction in growing season precipitation imposed as part of the Extreme Drought in Grasslands Experiment (EDGE), Carroll et al. 2021). Post-drought recovery was also monitored in each grassland. Because the same experimental plots were sampled throughout the entire 10-year period, including control plots, we can now directly compare the impact of these two droughts as well as recovery from them with consistent site-specific ANPP context (mean and interannual variability). Such context was previously unavailable in past assessments of these grassland responses to drought, so long term averages from areas within the same larger research sites were used as comparisons (Knapp et al. 2015; Griffin-Nolan et al. 2018a, b; Carroll et al. 2021). As a result we can now revise past estimates of drought sensitivity by using the data collected from the same exact plots over 10 years rather than data collected from various locations and potentially with different methods of ANPP estimation within the larger sites.

Our goals were to: 1) compare the magnitude of ANPP responses to natural *vs*. experimental drought in each of four grassland types (a C_4_ shortgrass prairie, a C_3_/C_4_ mixed-grass prairie, a C_4_ mixed-grass prairie and a C_4_ tallgrass prairie), 2) determine if the relative sensitivity to drought among grasslands varied by drought type, and 3) assess post-drought recovery of ecosystem functioning across these grasslands. We predicted that four years of extreme growing season precipitation reductions (the experimental drought) would impact ANPP more than a 1-year natural drought, and that post-drought recovery in ANPP would be delayed with longer-duration precipitation reductions (Oesterheld et al. 2001; Sala et al. 2012; Hoover et al. 2014). However, we expected that the relative sensitivity to drought (*i.e.,* productivity reduction per unit reduction in precipitation) among these grasslands would not differ between the one-year natural and four-year simulated droughts imposed (Huxman et al. 2004; Sala et al. 2012; Knapp et al. 2015). In other words, although the magnitude of drought impacts on these grasslands might vary by drought type and duration, the relative responses among the grasslands would be consistent.

## Materials and methods

### Site descriptions

**Fig. 1:**
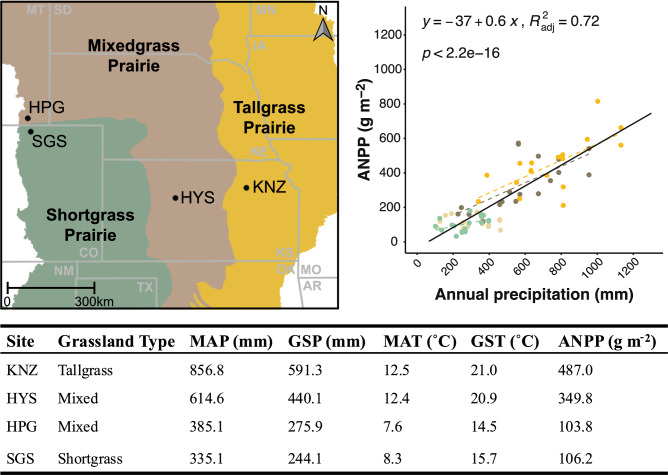
Site locations, characteristics, and ANPP sensitivity to annual precipitation at each of the four grasslands. Sites varied in their sensitivity of ANPP to interannual variability in precipitation (top right), shown by dashed lines for each site that showed at least marginally significant slopes (KNZ—gold, HYS—tan, and SGS—green; Table S1) and the solid black line showing the linear regression across all four sites. Climate characteristics of each site (bottom table) are long-term averages (1990–2020) for ambient mean annual precipitation (MAP), growing season (April 1– Sept. 15) precipitation (GSP), mean annual temperature (MAT), and growing season temperature (GST) (see Tables S2-3 for 2012–2021 annual MAP, GSP, MAT, and GST as well). ANPP values for each site are the 10-year average (of ambient plots) for each site over the course of the study (2012–2021). Map shows approximate site locations of each grassland in the United States, with state borders shown in gray lines and state labels in gray text (*MT* Montana, *SD* South Dakota, *MN* Minnesota, *CO* Colorado, *NE* Nebraska, *IA* Iowa, *KS* Kansas, *MO* Missouri, *NM* New Mexico, *OK* Oklahoma, *TX* Texas, and *AR* Arkansas; adapted from Carroll et al. 2021)

The four Central United States grasslands studied represent the major perennial grassland types in the Great Plains. Mean annual precipitation (MAP) at the sites increased from west to east by ~ 500 mm, with ANPP increasing similarly from ~ 100 g m^−2^ to nearly 500 g m^−2^ (Fig. 1). Interannual variability in precipitation is generally strongly correlated with ANPP both within and across these grasslands, typical of broader patterns across the Great Plains (Fig. 1, Sala et al. 1988). Indeed, the slope of the relationship between MAP and ANPP across four sites during our study years (Fig. 1) was identical to that previously reported in Sala et al. (1988). The northern mixed grass prairie (HPG) at the High Plains Grassland Research Center in Cheyenne, Wyoming is codominated by the C_3_ *Pascopyrum smithii* mixed with C_4_ *Bouteloua gracilis*, with the latter species being the dominant grass at the northern shortgrass steppe (SGS) in the Central Plains Experimental Range near Nunn, Colorado. The warmer, more productive grasslands near Hays, Kansas and Manhattan, Kansas included a southern mixed grass prairie (HYS; at the Hays Agricultural Research Center) and an annually burned tallgrass prairie (KNZ; at the Konza Prairie Biological Station), both C_4_-dominated. Dominant species of the southern mixed grass prairie (HYS) include *Bouteloua curtipendula, Schizachyrium scoparium,* and *P. smithii* and dominant tallgrass (KNZ) species were *Andropogon gerardii* and *Sorghastrum nutans*. The two mixed grass prairie sites were lightly grazed in the two years prior to our study (2010–2011) while the tallgrass and shortgrass steppe study plots had not been grazed by domestic herbivores for > 10 years prior to the start of this study in 2012. No sites were grazed over the course of the experiment (2012–2021).

### Contrasting the natural vs. experimental drought

In 2012, much of the United States experienced severe drought conditions including all four of the grassland sites included in this analysis (Cook et al. 2014). This pan-continental drought was a relatively rare event in both its extent and severity, but greenhouse gas forcing is expected to increase the occurrence of such droughts in the future (Cook et al. 2014). At the four EDGE sites, annual precipitation was reduced by nearly 40% in 2012 which in turn reduced ANPP at each site by 25–50% compared to long-term averages (LTA) from nearby areas (Knapp et al. 2015). The legacy effects of this drought (Vilonen et al. 2022) continued to affect ANPP at some sites into 2013 (Griffin-Nolan et al. 2018a, b). The EDGE plots were constructed starting in 2012, but the manipulated drought treatments did not start until 2014 at each site and continued through 2017. EDGE was designed as a coordinated distributed experiment (Fraser et al. 2013) with identical methods employed at the four sites. We used data collected from EDGE beginning in 2012 to monitor responses to the natural drought and one year of recovery in 2013. We then compared those responses to the manipulated drought treatments from 2014 to 2017, and similarly monitored recovery from the four year experimental drought through the four years post-drought (2018–2021).

EDGE compared two drought treatments, both causing a ~ 66% reduction in growing season precipitation, but imposed in slightly different ways. The “chronic treatment” reduced all rain events by 66% over the entire growing season and an “intense treatment” eliminated all rain events, but for a shorter portion of the growing season. One replicate of each treatment was randomly assigned into one of three plots in each of the 10 experimental blocks at each site. Thus, each block consisted of three 6 × 6 m plots (n = 30 independent plots per site), two of which were covered with partial or full transparent roofs for all or part of the growing season and one with the shelter structure but no roofs present, to act as an infrastructure control (with ambient precipitation). Each 36 m^2^ plot was hydrologically isolated from its surroundings using plastic sheeting buried belowground and aluminum flashing partially buried to prevent aboveground overland water flow. Plastic roofs were installed and removed each year such that treatments were imposed from ~ April 1 through mid-September. Previous studies with these types of shelters have found little effect of micrometeorological differences caused by the roofs on plant responses (Loik et al. 2019), with the largest differences in air temperatures happening at night. Photosynthetically active radiation (PAR) can be reduced by shelters, though the implications on plant physiological responses overall are minimal (Yahdjian and Sala 2002; English et al. 2005; Signarbieux and Feller 2012; Loik et al. 2019). Therefore, our climate variables for VPD and temperature (described below) are from outside of roofing structures, but should be comparable to levels under roofs as well. Further details on drought treatments and experimental design can be found in Carrol et al. (2021). After the 4-year experimental drought treatments ended, we monitored post-drought ANPP for an additional four years (2018–2021).

Carroll et al. (2021) analyzed the effects of the two types of drought treatments on ANPP during the experimental drought period (2014–2017) and found that, while droughted plots differed significantly from ambient plots, the two drought treatments (chronic *vs.* intense drought) seldom differed significantly from each other. Thus, we combined them into one experimental drought treatment for this analysis. By combining the drought treatments, we were left with n = 10 ambient plots per site and n = 20 drought plots for site, rather than the original n = 10 of each (ambient, chronic, and intense drought). When directly comparing or combining responses of ambient and droughted plots (such as percent reductions or drought sensitivity – see below), we used the one ambient plot and the average of the two drought plots from the same block.

### Climate variables

We compiled climate data from a variety of sources for this analysis. During the experimental drought period (2014–2017), ambient precipitation was measured at each site with tipping-bucket rain gauges. Inputs into the droughted plots were calculated based on estimates of reductions from drought shelter designs and were adjusted as needed using soil moisture data to account for any rainfall blowing into plots, temporary roof damage, etc. During pre-treatment and post-drought recovery periods (2012–2013 and 2018–2021), precipitation inputs were determined from a combination of site rain gauges when available, the nearest weather station at the research sites, or the nearest National Oceanic and Atmospheric Administration (NOAA) stations. To assess long term climate averages, we combined our experimental climate data with earlier NOAA records (1990–2011), using daily precipitation data from the closest NOAA station gap-filled as needed with data from surrounding stations (90% of observations were from stations within 15 km of the sites with the 10% used for gap-filling taken from further NOAA stations; Menne et al. 2012a, b). Temperature and vapor pressure deficit (VPD) data were retrieved using PRISM for all years (1990–2021, PRISM Climate Group 2024). All climate data were collected and analyzed at the site-level, and we defined the growing season as April 1–September 15 for all sites and all variables.

### ANPP responses

ANPP was measured at each site at the end of each growing season by harvesting all aboveground biomass within 20 × 50 cm quadrats (typically three per plot, although occasionally these precision replicates were reduced to two or rarely one – 11 and 4% of all plots, respectively – only during the 4-year recovery period). Harvested quadrats were randomly located within subplots dedicated to destructive sampling and flagged to avoid resampling in consecutive years. Biomass was sorted into C_3_ and C_4_ grasses, forbs, and woody (if present) biomass, as well as previous years’ dead material, before being dried at 60˚C for 48 h and weighed to the nearest 0.01 g. Ten-year ANPP averages were calculated from the control (ambient precipitation) plots at each site from 2012 to 2021 (n = 30 plots per site, except for KNZ where plots were not yet established in 2012, so we used n = 5 plots from a nearby area with similar soils and topography). From 2014 to 2021, we used only the ambient plots (n = 10 plots per site) that had not be exposed to experimental drought treatments in the calculations of ANPP LTA’s. In total, this allowed us to estimate ANPP LTA’s with > 500 samples collected across all site’s ambient plots over all years. As noted above, this provided us with a more precise estimate of ANPP and its interannual dynamics compared to previous analyses, which had to rely on ANPP data from past studies that varied in collection methods, location, and sampling intensity (Knapp et al. 2015).

To quantify drought responses, we calculated the proportional (%) reduction in ANPP in droughted vs. ambient plots for each year (2014–2017) of the experimental drought (n = 10 per site – calculated as the average reduction of ANPP in the two drought plots compared to the one ambient plot in each block). Because all plots, including ambient plots, were subject to the natural drought in 2012, we calculated the proportional reduction in ANPP in response to the 2012 drought relative to the mean ambient ANPP for the 9 years after this drought. Similar comparisons were made during the recovery years – in 2018–2021 comparing droughted vs. ambient plots, and in 2013 comparing ANPP at each site to the ambient ANPP average.

Because control (ambient) plots were not available for the 2012 drought, we statistically assessed ANPP responses to drought and recovery post-drought separately for the natural and experimental droughts (see below). Further, to better match time scales, we assessed the ANPP response to the experimental drought in two ways: focusing on just the first year of drought (to match the duration of the natural drought) as well as the average ANPP response across the entire 4-year experimental drought period. Recovery periods were similarly assessed (1-year recovery period from the natural drought, and both 1-year and 4-year-averaged recovery from experimental drought). We evaluated time (Year) as a variable in the 4-year drought and recovery periods, and although ANPP did differ significantly among years, there were no clear temporal patterns for either drought duration or post-drought recovery (see Fig. 3a below). Thus, we focused on the average 4-year response and recovery at each site.

Finally, because previous estimates of relative drought sensitivity were determined by calculating the reduction in ANPP per unit change in precipitation (g m^−2^ mm^−1^; Huxman et al. 2004; Knapp et al. 2015) we calculated drought sensitivity in a comparable way, as productivity (ANPP) changes divided by precipitation (PPT) changes.

For natural drought:

$$\frac{{ANPP}_{2012}-{ANPP}_{2013-2021\ average}}{{PPT}_{2012}-{PPT}_{30-yr\ average}}$$

And for experimental drought:

$$\frac{{ANPP}_{drought\ plot}-{ANPP}_{ambient\ plot}}{{PPT}_{drought\ treatments}-{PPT}_{ambient}}$$

where long-term averages (ANPP 2012–2021) for each site were used as “ambient” values for the natural drought period (2012–2013, when no undroughted plots were available) and the actual observed ambient and drought plot measurements for the experimental drought period (2014–2021). These estimates of sensitivity were also used to compare directly to previous estimates of drought sensitivity according to the Huxman-Smith model (Huxman et al. 2004).

### Statistical analysis

**Fig. 2:**
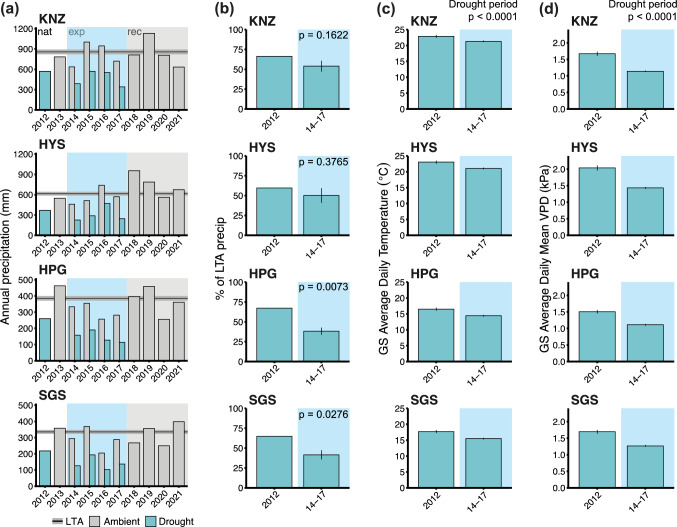
Drought conditions during the natural (2012) and experimental (2014–2021) drought periods (also see Table S2-S4 for yearly climate variables at year site). **a** Annual precipitation (mm) at each site for the natural drought years in 2012 and recovery in 2013 (white background), during the experimental drought years (blue background) in control/ambient plots and droughted plots, and for experimental recovery years (2018–2021; gray background shading). Each site has horizontal lines showing the 30-year MAP with ± 1 SE gray shading. **b** Percent reductions in precipitation compared to long-term averages (LTA) for each site. Reductions for the natural drought year were compared to the average across experimental drought years using one-sample t-tests (Table S5). **c** Average daily temperatures during the growing season (GS) were significantly higher for the natural drought across all sites. **d** Daily mean VPD’s during the GS were also higher across all sites. Simple linear regressions and ANOVAs were used to assess effects of site and drought period (natural vs. experimental) for temperature and VPD (not including interaction effects since they were non-significant, see Table S5 for details)

All statistical analyses were performed in R version 4.3.2 (R Core Team 2023). We first compared climate variables (precipitation, air temperature, and VPD at the site-scale) between the natural drought year (2012) and averaged across all experimental drought years (2014–2017). Since precipitation varies considerably across years (Fig. 2a), we calculated each year’s annual precipitation in terms of the percent of the LTA precipitation for each site. We then used the % LTA precipitation in 2012 as the reference value in a one-sample t-test compared to the average % LTA precipitation from the four years of experimental droughts (using only the precipitation from the drought treatment reductions; Fig. 2b). We also compared daily air temperatures and VPDs during the natural and experimental drought periods using the average daily temperature or VPD during the growing season. We created simple linear regressions using the lme4 package in R (Bates et al. 2015) and the car package Anova function (Fox and Weisberg 2019) with daily temperature or VPD as the response variable and site and drought type (natural or experimental) as fixed effects, and since their interaction was significant, we used the emmeans package (Lenth 2024) to assess pairwise comparisons for drought type differences within each site (Table S5).

Measures of ANPP (g m^−2^) were analyzed at the plot level (n = 20 drought and 10 ambient plots per site) and ANPP sensitivity (g m^−2^ mm^−1^) at the block level (n = 10 per site because we combined the two drought plots with the one ambient plot in each block, as explained above). Blocks and plots remained consistent throughout the 10-year period, so any analyses of differences in multiple years included plot or block as a random effect to account for repeated measures in the same experimental units over time and additionally included a “time” or “period” effect with plot or block when plots were measured across treatments (e.g., when comparing plots across the natural and experimental drought periods). Before analyzing any ANPP data, we checked for and removed ANPP outliers within groups for each site and each year since substantial variability in annual and site ANPP is expected. We used the interquartile range (IQR) method for identifying outliers and only removed any observations that were defined as extreme (± 3•IQR below the first quartile or above the third) and only if no clear ecological explanation existed for those ANPP fluctuations (such as large forbs driving increased biomass in particular plots). Of the ~ 1200 plots harvested for ANPP across all sites and years, only 20 (~ 1.7%) were determined to be outliers and dropped before analysis.

Differences in ANPP were assessed using repeated measures mixed effects ANOVAs within each period: the natural drought and recovery (2012 vs. 2013), during the experimental drought (2014–2017), and in the four years of recovery following experimental drought (2018–2021; Table S6). Within each of these periods, we fit separate linear regressions using the lmer function of the lme4 package and anova function of the lmerTest package (Bates et al. 2015; Kuznetsova et al. 2017) with ANPP (g m^−2^) as the response variable. Site, Year, experimental drought treatment, and their interactions were the fixed effects (except in 2012–2013 where only Site and Year were fixed effects as no experimental drought treatments had begun yet) and plot as a random effect to account for repeated measurements in the same plots over time. In 2012, experimental plots at KNZ were not yet set up, so we used estimates of ANPP from a nearby site, though our results do not change whether KNZ is analyzed along with the other sites in 2012 or on its own. For all periods, the interactions between Site, Year, and Treatment (when included) were significant, so we followed ANOVAs with pairwise comparisons again using the emmeans function (Table S6; Lenth 2024). Pairwise comparisons of 2014–2018 annual ANPP estimates allowed us to identify significant reductions from the experimental drought treatments compared to ambient precipitation plots, but in 2012–2013, all plots were under the same ambient conditions – so we additionally compared 2012 and 2013 ANPP estimates to the long term average (LTA) ANPP for each site using simple linear regressions with Site and Period (2012 or 2013 alone as one period and the other 9 years as the second period) as fixed effects, and again followed with pairwise emmeans comparisons to check for differences within each Site.

We calculated ANPP sensitivity for 2012 and during the 2014–2017 experimental droughts as the reduction in ANPP (g m^−2^) divided by the reduction in precipitation (mm), with 2012 ANPP and precipitation compared to site LTA’s and 2014–2017 ANPP and precipitation in droughted plots compared to ambient plots in the same blocks. We compared ANPP sensitivity during the natural drought to both the first year of the experimental drought (2014) and across all years of the experimental drought (2014–2017) using independent repeated measures ANOVAs again with ANPP sensitivity as the response variable and fixed effects for Site and the drought type (natural or experimental). For both, we included Block as the random effect to account for repeated measures and for the comparison across drought years also included a Block–Period effect to account for plots being compared in one natural year vs. across multiple experimental drought years. In both 2012 vs. 2014 and 2012 vs. the 2014–2017 average, the interactions between Site and drought type were not significant, so we re-fit models without the interactive effect and were most interested in drought type effects over all sites (Table S8).

To identify any legacy effects of drought, we evaluated the recovery years (2013 and 2018–2021) independently. As described above, we used a simple linear regression and ANOVA with pairwise comparisons to compare 2013 ANPP to the ANPP across all nine other years (Table S7). After the experimental drought (in 2018–2021), we compared ANPP in previously-droughted plots with ambient plots, both in the first year of recovery and averaged across all recovery years. We evaluated recovery with ANPP as the response variable and Site, Treatment, and their interaction as fixed effects in simple linear regressions and ANOVAs followed again by emmeans pairwise comparisons to check for differences in treatment effects within the different sites (Table S9). For all analyses, model assumptions were assessed graphically and response variables transformed as needed to best meet assumptions for normality and equal variance. All significance was assessed at α = 0.05. Full results are included in the Supplemental Material, including types of transformations on response variables and output of all t-tests, ANOVAs, and pairwise comparisons.

## Results

### Drought characteristics

In 2012, the natural drought reduced growing season and annual precipitation by 35–40% for all sites (Fig. 2b, Knapp et al. 2015). Growing season precipitation reductions imposed by the experimental drought treatments were targeted at 66%, but actual reductions relative to each site’s long-term precipitation records varied from 53 to 71% below average across the four years (or 46–61% reductions in annual precipitation compared to MAP for each site; Fig. 2b). Thus, both the magnitude of precipitation reductions and their duration (4-years *vs*. 1-year) were greater in the experimental than the natural drought. Limited plot-level soil moisture data were available only during the experimental drought, and these data confirm the effectiveness of precipitation reductions in reducing soil moisture levels (Fig. S1). Average growing season temperatures in 2012 were ~ 2.0˚C warmer than during 2014–2017 (ranging from + 1.59˚C at KNZ to + 2.22˚C at SGS) and 2012 growing season VPD was also significantly higher (mean = 0.49 kPa, ranging from 0.4 kPa at HPG to 0.61 kPa at HYS) than during the experimental drought years (Fig. 2b–d). Finally, the seasonality of the natural and experimental droughts varied as well. While the seasonal timing and duration of the 4-year experimental drought was consistent across all the grassland sites (treatments imposed from ~ April 1- September 15), the seasonality of the 2012 drought varied substantially across the sites (Fig. S2). For the drier grasslands (HPG and SGS), the 2012 drought was most severe early in the year (March–May) whereas at HYS, the 2012 drought occurred primarily during the summer months (June–August), similar to the timing of the experimental drought (Fig. S2). The KNZ site was intermediate between these two patterns.

### ANPP responses

**Fig. 3:**
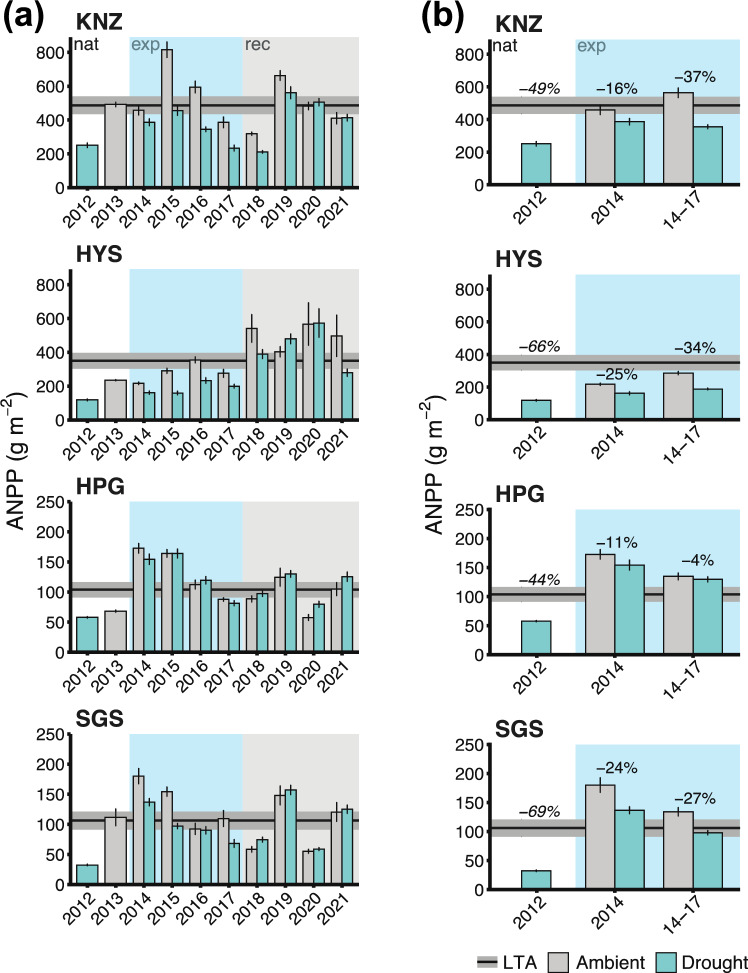
**a** ANPP (mean ± 1 SE) at each site across all 10 study years. Background shading indicates the drought periods: white – natural drought/recovery years, light blue – experimental drought years, and gray – experimental recovery years. 10-year average ANPP for each site is shown with the horizontal black line (mean) with gray shading (± 1 SE). **b** The natural vs. experimental drought ANPP responses and reductions (%) compared to either the 10-year ANPP average (horizontal lines and italicized numbers) for 2012 (white background) or to ambient plots during the drought experiment years (blue background; for full comparisons and statistics, see Tables S6-S8 – note that 2012 bars’ percent reductions (**b**) are compared to each site’s 10-year averages but statistics compare 2012 to 2013–2021 9-year averages only)

In general, all drought years (2012 and 2014–2017) reduced ANPP at all four sites with the C_3_ dominated HPG site typically responding less than the C_4_ grasslands (Fig. 3). The 2012 pan-continental drought reduced ANPP the least (44%) at the C_3_ dominated HPG site and the most (69%) at the SGS site (Fig. 3). These estimates exceeded the 25–55% reductions previously reported by Knapp et al. (2015), reflecting our updated estimates of ambient (non-drought) ANPP available for each site instead of the previous method of using long-term ANPP data from outside of the experimental area. During the first year of the experimental drought (2014), ANPP reductions during the experimental drought were much less than the 2012 drought – varying from 11 to 25% across the four sites. Even after four years of simulated drought, ANPP reductions ranged from only 4% at HPG to ~ 40% at KNZ. Thus, although ANPP at the C_3_ grassland (HPG) responded significantly (Site–Period interaction F_3,203.09_ = 11.992, p < 0.0001 and HPG pairwise t_148_ = − 5.757, p < 0.0001; Table S7) to the natural drought (44% reduction), we did not observe overall significant reductions at this site between ambient and droughted plots during the experimental drought years (Fig. 3, Table S6). In contrast, the response to experimental drought was significant for most years in all of the C_4_ grasslands, but reductions were generally less than in 2012 (Fig. 3b, Table S6). Thus, contrary to our expectation that multi-year experimental drought would impact ANPP more than a one-year natural drought, we found that ANPP was reduced to a much greater extent during the natural drought.

### Relative ANPP sensitivity to natural vs. experimental drought

**Fig. 4:**
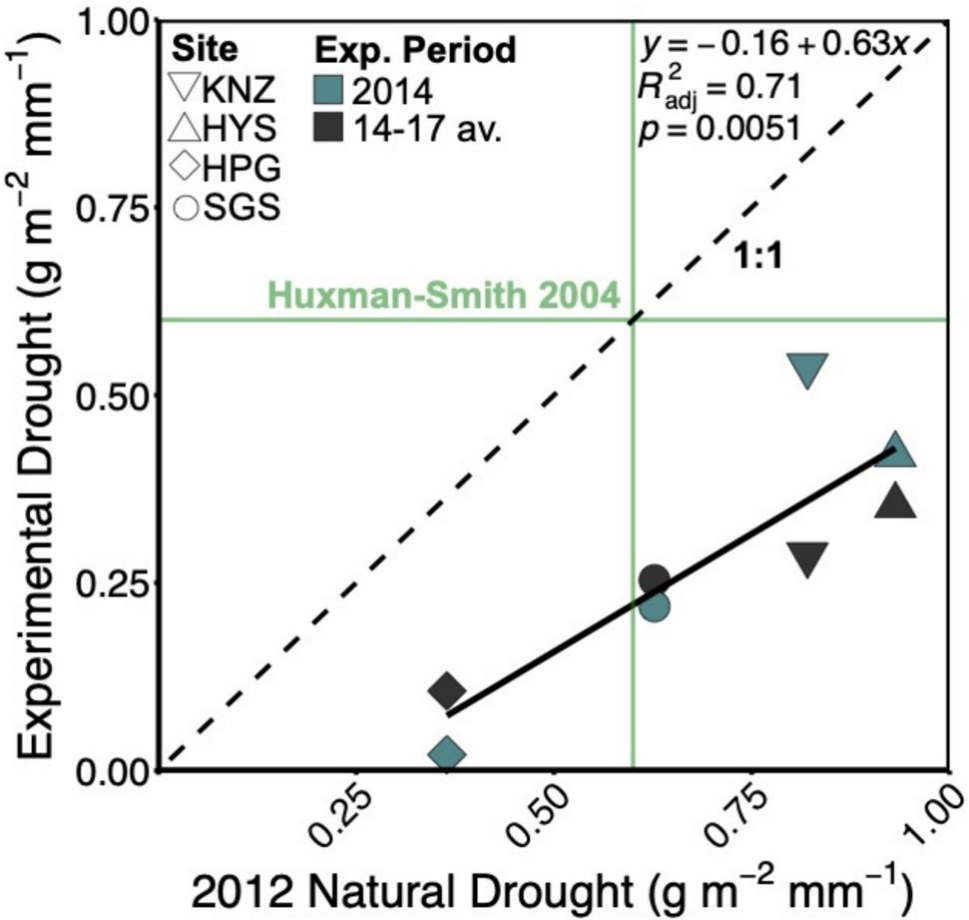
ANPP sensitivity (g m^−2^ mm^−1^) to natural and experimental drought for each site comparing sensitivity to the natural drought and the experimental drought for both the first year of experimental drought (black points) and the average of the four experimental drought years (blue points). For our sites, ANPP sensitivity is expected to be no more than 0.6 according to Huxman et al. (2004). For the experimental drought, all sites fall under the max sensitivity (green horizontal line), but for the natural drought, three of the four sites had greater sensitivity than previously reported (green vertical line)

Because the four grasslands varied significantly in both ANPP and precipitation, we used a standardized metric, g m^−2^ reduction in ANPP per mm reduction in precipitation, for our assessment of drought sensitivity. Ecosystems, and especially grasslands, are known to differ in their sensitivity to drought (Huxman et al. 2004; Cherwin and Knapp 2012; Knapp et al. 2015; Zuo et al. 2022; Jaman et al. 2022), and the four grasslands we studied did indeed differ in drought sensitivity (g m^−2^ mm^−1^) in response to the natural drought, the first year of the experimental drought (F_1,34.945_ = 49.1447, p < 0.0001; Table S8), and for the average of all experimental drought years (F_1,64.704_ = 68.200, p < 0.0001; Table S8). As expected from comparisons of absolute and relative ANPP responses (Fig. 3), drought sensitivity was consistently lower in response to the experimental drought (1st year and over all 4 years) than the natural drought (F_1,6_ = 18.4066, p = 0.0051; Fig. 4) but the relative ranking of drought sensitivity among the sites did not differ markedly (Fig. 4). In general, the C_3_-dominated grassland was the least sensitive to drought and the two warmer C_4_ grasslands were more sensitive. Of note, the drought sensitivity estimates for the simulated droughts were all within the range reported previously (Huxman et al. 2004) based on long-term ANPP-precipitation relationships. In contrast, the estimated drought sensitivity for the natural drought (which combined reduced precipitation and elevated temperatures and VPD) was greater than previously reported for three of the four grasslands (Fig. 4).

### Post-drought recovery

**Fig. 5:**
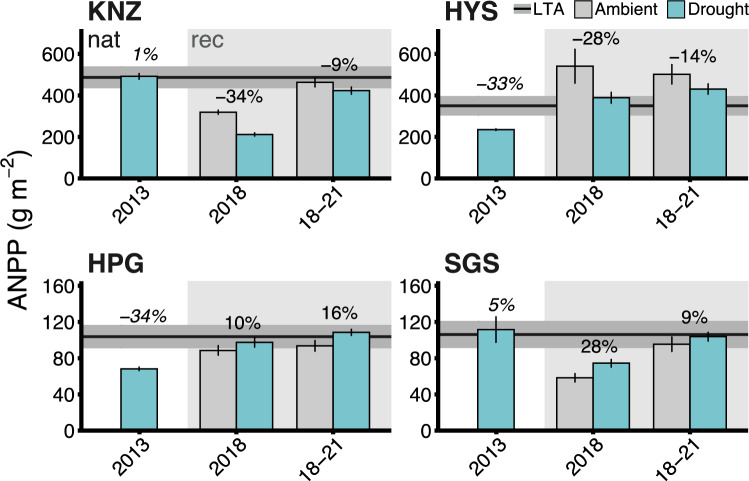
ANPP (mean ± 1 SE) responses and percent reductions or increases during recovery after natural drought (2013 recovery year from 2012 natural drought – white background) and the experimental drought (2014–2017 drought with recovery in the first year (2018) or averaged across all recovery years (2018–2021) – gray background). Horizontal black lines show the 10-year ANPP averages for each site (± 1 SE gray shading). Percent reductions are shown comparing 2013 ANPP to the 10-year average (horizontal lines and italicized numbers) and 2018–2021 reductions in droughted plots compared to control/ambient plots (for full comparisons and statistics, see Table S9 – note that plots show percent reductions for each site vs. their 10-year averages but statistics compare 2013 to only 2012 and 2014–2021 9-year averages)

Patterns of drought recovery were generally inconsistent when comparing the natural vs. experimental droughts. In the recovery year after the 2012 natural drought, only SGS showed significant changes in ANPP compared to long-term site averages with increased ANPP (Site–Period interaction F_3,182.70_ = 4.2728, p = 0.0065 and pairwise t_130_ = 3.143, p = 0.0021; Table S7), compared to the long-term site averages (Fig. 5), while ANPP in the year after drought at KNZ, HYS, and HPG did not differ from their long-term means. In contrast, ANPP at KNZ and HYS remained reduced in the first recovery year after the 4-year experimental drought (Site–Treatment interaction F_3,111_ = 7.3685, p = 0.0002; KNZ pairwise t_111_ = 3.780, p = 0.003; HYS pairwise t_111_ = 2.338, p = 0.0212; Table S9), whereas ANPP at SGS was increased (SGS pairwise t_111_ = − 2.118, p = 0.0364; Table S9) and at HPG did not differ between previously droughted and ambient plots (HPG pairwise t_111_ = -0.725, p = 0.4701; Table S9). When the entire 2018–2021 recovery period was assessed, only HPG shows marginal evidence of post-drought legacies impacting ANPP (Site–Treatment interaction F_3,112.01_ = 3.0674, p = 0.0309; HPG pairwise t_113_ = − 1.972, p = 0.0511; Table S9), possibly driven by ANPP responses in 2020 (Fig. 3) which was the driest recovery year (Fig. 2). However, the lack of response during drought in this site makes this response difficult to attribute to the experimental drought treatment.

## Discussion

Comparisons of ecological responses to natural meteorological anomalies vs. those simulated via controlled experiments can be challenging, particularly across multiple sites, but insights can still be gained (*e.g.,* Peñuelas et al. 2007; Sandel et al. 2010, Yuan et al. 2017). Here, we took advantage of 10 years of drought research in four native grasslands that spanned a broad precipitation gradient and differed in species composition and many key plant traits (Griffin-Nolan et al. 2018a, b; Carroll et al. 2021). Each of these grasslands were exposed to a natural, pan-continental drought in 2012 followed two years later by an experimental drought (four years of growing season precipitation reduction). Key advantages of this comparison are that the natural drought was of similar severity for all grasslands (Knapp et al. 2015) as was the experimental drought (Carroll et al. 2021), the experimental drought was imposed identically at all sites, post-drought recovery was measured for both droughts at all sites with recovery periods equal to drought duration, and the focal response variable (aboveground net primary productivity, ANPP) was measured consistently and from the same plots over the entire 10-year period. Of course, there are also multiple caveats that must be acknowledged when comparing responses to these two droughts. Primary among these are the obvious differences in drought duration (one vs. four years), the high air temperatures and VPD that co-occurred with the natural drought but were not present during the experimental drought, and the more subtle differences in the seasonality of the droughts. Indeed, drought duration, atmospheric demand, and the seasonal pattern of water limitation during drought are all expected to impact ANPP responses and potentially post-drought recovery (Felton et al. 2020; Hajek and Knapp 2022; Gamelin et al. 2022; Wright and Collins 2024).

The key insights from this study are, first, that the 1-year 2012 drought reduced ANPP more than the experimental drought – and this was true for both the first year of the simulated drought and when drought impacts were averaged over the entire 4-year drought period (Fig. 3). Note that apart from the C_3_ dominated HPG site, there were some individual years for each of the C_4_ grasslands when differences in ambient vs. droughted plots were of a similar magnitude to the 2012 natural drought, but these were the exceptions rather than the rule (Fig. 3). This pattern occurred despite the experimental drought imposing greater reductions in precipitation inputs than the natural drought. This general conclusion was also evident when comparing previous independent analyses of these droughts (Knapp et al. 2015; Carroll et al. 2021), but the degree of divergence in ANPP reductions is now greater based on the decadal ANPP data available at each site. For example, Knapp et al. (2015) reported that ANPP was reduced by ~ 25–50% from the 2012 drought, but our revised estimates are ~ 45–75% ANPP based on more accurate estimates of mean ANPP for each site (Fig. 3). Revised estimates of the 2012 drought impact differed most strongly at the HYS and SGS sites, while the ANPP reductions at the HPG and KNZ sites were similar. In contrast, estimates of ANPP reductions to the experimental 4-year drought (Fig. 3) ranged from very little reduction at HPG to ~ 40% ANPP reductions at KNZ, with these broadly similar to those reported previously (Carroll et al. 2021). Overall, these results support previous concerns that simulating drought via precipitation reductions alone can underestimate natural drought impacts because concurrent increases in air temperature and VPD are also not imposed (De Boeck and Verbeeck 2011; Novick et al. 2016; Kröel-Dulay et al. 2022; Wright and Collins 2024). Based on past complementary analyses for this grassland region, it is likely that increases in VPD will impact productivity more than warmer temperatures (Mowll et al. 2015; Konings et al. 2017).

It is worth noting that during the experimental drought, proportional reductions in graminoid ANPP were generally greater than total ANPP at HYS and HPG and less than total ANPP at SGS (and during recovery, we saw similar trends for these sites, Fig. S3), reflecting potential shifts in species composition during the 4-year simulated drought. When comparing proportional reductions in biomass separated by functional groups (C_3_ or C_4_ grasses, forbs, and woody plants when present), we noted greater reductions in C_4_ grasses then C_3_ grasses at the three C_4_-dominated grasslands (KNZ, HYS, and SGS) and proportional increases in forb biomass at SGS and HPG in the last year of experimental drought (Fig. S4). However, previous analyses of community composition data by Griffin-Nolan et al. (2019) reported community shifts only from C_4_ to C_3_ grasses at HYS and SGS over the course of the four-year experimental droughts, with no observable effect of the experimental drought treatments at KNZ or HPG. During the single-year natural drought, there were small proportional reductions in C_3_ grasses and forbs from 10-year functional group biomass averages that tended to be greater than reductions in total ANPP at HPG and SGS. However, at HYS, C_3_ grasses increased modestly compared to 10-year biomass averages – which is not unexpected as this grassland was the only site where the 2012 natural drought reduced summer precipitation more than spring precipitation (Hajek et al. 2024). At KNZ, 2012 biomass data was only available as graminoid and total ANPP, and here both were reduced equally. The experimental drought had the greatest precipitation reductions in the summer months (Fig. S2), so differences in functional group responses to different timing of drought at not unexpected (Knapp et al. 2020; Hahn et al. 2021; Hajek et al. 2022).

The second insight is that, despite the experimental drought underestimating the magnitude of ANPP reductions, relative differences in drought sensitivity among grasslands were similar when comparing the natural vs simulated drought among the four sites. The Huxman-Smith model of ecosystem sensitivity to changes in precipitation predicted that the greatest sensitivity to drought, and the greatest variability among ecosystems in drought sensitivity, will occur where MAP < 1000 mm (Huxman et al. 2004), a pattern confirmed by Mauer et al. (2020). We observed a similar relative ranking of drought sensitivity among the 4 sites (all less with < 1000 mm MAP) based on both the natural and experimental drought (Fig. 4), with the C_3_ dominated grassland (HPG) consistently the least sensitive. Many C_3_ grasslands in North America rely on winter and early spring soil moisture and can be relatively insensitive to reductions in precipitation during the summer months (Frank 2007; Knapp et al. 2015, 2020).

It is also worth noting that the estimates of drought sensitivity from the 4-year simulated drought were quantitatively similar to the Huxman-Smith estimates (Huxman et al. 2004) whereas this sensitivity metric was generally higher for the natural drought (Fig. 4). The underestimation in sensitivity to simulated drought compared to the natural drought is likely because both the Huxman-Smith and simulated drought estimates of drought sensitivity are primarily driven by changes in precipitation. However, the natural drought additionally involved increased air temperatures and VPD (Fig. 2). Indeed, differences in this sensitivity metric between the simulated and natural drought diverged most in the two most mesic grasslands – consistent with prior analyses indicating that productivity in more mesic ecosystems will be more responsive to changes in VPD than in drier ecosystems, which are driven more by soil moisture (Novick et al. 2016; Kannenberg et al. 2024).

Finally, we found it difficult to draw any insights from comparisons of post-drought recovery patterns after the 1-year vs 4-years drought. Previously, Griffin-Nolan et al. (2018a, b) reported that legacy effects from the 2012 drought had both positive and negative legacy effects on productivity post-drought. At the C_3_ dominated site (HPG), ANPP did not recover in the first year post-drought (suggesting a negative drought legacy) whereas the C_4_ sites fully recovered, with ANPP at KNZ showing a slight positive drought legacy impact (higher ANPP in drought plots vs. ambient plots) in 2013. Based on our revised estimates of mean ANPP for each site, similar post-drought recovery patterns were observed in our reanalysis (Fig. 5) although the positive legacy of the 2012 drought at KNZ was no longer evident. Comparing the first year of recovery after the 4-year simulated drought (2018) to the natural drought is complicated by substantial differences among sites in annual precipitation in 2018 vs. 2013 (Fig. 2). In the first year after the 4-year simulated drought, post-drought legacy effects were negative at KNZ and HYS, positive at SGS and not evident at HPG, with all sites displaying largely idiosyncratic patterns of recovery throughout the 4-year post-drought period (Figs. 3, 5). The general conclusion of Griffin-Nolan et al. (2018a, b) that there is evidence for both positive and negative drought legacy impacts among the four grasslands is also supported by recovery dynamics after the experimental drought. However, it is clear that the determinants of drought recovery of ecosystem function are varied, likely site specific, and will require additional study to more fully understand this aspect of ecosystem resilience (Sala et al. 2012; Vilonen et al. 2022).

In conclusion, our results underscore the limitation of manipulating precipitation and soil moisture as an approach for quantifying drought impacts on ANPP in ecosystems. Previous authors have suggested that passive precipitation reduction shelters may underestimate drought impacts by 53% (Kröel-Duley et al. 2022); our results were consistent with this magnitude of underestimation. Further, with trends of increasing VPD associated with warming, the magnitude of such underestimates may increase in the future. However, there are likely many interactions between decreasing soil moisture and increasing VPD during drought that required further exploration (Novick et al. 2024). It is possible, for example, that in dry ecosystems where soil moisture is extremely low during drought and primary producers become physiologically inactive, the impact of increasing VPD may be minimal relative to more mesic ecosystems (Novick et al. 2016; Xu et al. 2023; Kannenberg et al. 2024). If future experiments can manipulate VPD and soil moisture separately (Wright and Collins 2024), or parse them independently (Roby et al. 2020; Kannenberg et al. 2024), estimates of both the independent and combined impacts of increased VPD and decreased soil moisture during drought will be possible. However, even with the shortcoming of experimental manipulations underestimating the magnitude of drought impacts, such experiments can still provide valuable insight into the relative responses of ecosystems to reduced soil moisture, which remains a key aspect of understanding droughts.

## Supplementary Information

Below is the link to the electronic supplementary material.

Supplementary file1 (DOCX 901 KB)

## Data Availability

Experimental data is available from the corresponding author on reasonable request. All climate data are publicly available at NOAA’s National Climate Data Center (www.ncdc.noaa.gov/) and the PRISM Climate Data website (www.prism.oregonstate.edu/).
