# Supplementary material for: Experimental drought consistently underestimates productivity responses to natural drought in four Central US grasslands
Source: Oecologia. 2025 Jun 19;207(7):104. doi: 10.1007/s00442-025-05746-9 (PMC12178983; doi:10.1007/s00442-025-05746-9)
Supplement: Supplementary file 1 — Supplementary file1 (DOCX 901 KB) [file 442_2025_5746_MOESM1_ESM.docx]

Supplemental Material: Experimental drought consistently underestimates productivity responses to natural drought in four Central US grasslands

Kathleen V. Condon^1*^, Charles J. W. Carroll^2^, Robert J. Griffin‑Nolan^3^, Ingrid J. Slette^4^, Kate D. Wilkins^5^, Melinda D. Smith^1^, and Alan K. Knapp^1^

^1^Department of Biology and Graduate Degree Program in Ecology, Colorado State University, Fort Collins, Colorado, USA

^2^Forest and Rangeland Stewardship, Colorado State University, Fort Collins, Colorado, USA

^3^Department of Biological Sciences, California State University, Chico, California, USA

^4^Department of Ecology, Evolution, & Behavior, University of Minnesota, St. Paul, Minnesota, USA

^5^Denver Zoo, Denver, Colorado, USA

*Corresponding author: [kathleen.condon@colostate.edu](mailto:kathleen.condon@colostate.edu)

**Figure S1.** Monthly soil moisture volumetric water content (VWC, %) averages at three of sites (KNZ, HYS, and HPG) from April to September 2014-2017, each year of the experimental drought. Colors correspond to treatments (gray = ambient conditions, blue = droughted plots). Sensor issues prevent the inclusion of SGS. Figure adapted from Carroll et al. 2021.

**Figure S2.** Seasonal patterns of precipitation in 2012 (natural drought) and 2014-2017 averaged (experimental drought) years. The 2012 natural drought reduced spring precipitation more than summer precipitation at all sites except for Hays while the 2014-2017 experimental drought years had the greatest precipitation reductions in summer.

**Figure S3.** **(a)** Natural vs. experimental drought responses of graminoid species only. **(b)** Natural vs. experimental drought recovery of graminoid species. Both figures show natural drought (white background shading) compared to the 10-year graminoid average ANPP (horizontal bar and ±1 SE shading) for our experimental drought plots (light blue or gray background shading) to ambient plots during the drought experiment and recovery years.

**Figure S4.** Responses in aboveground biomass for each function group (g m^-2^) during the 2012 natural drought and the first and last years of experimental drought (2014 and 2017). Bars show average biomass for each year (± 1 SE) with gray bars for control plots and blue for droughted plots. Horizontal lines represent each site’s 10-year average ANPP for each functional group (±1 SE shading), though averages at KNZ are based on 2013-2021 only as 2012 data was not divided into each functional group (hence also the exclusion of 2012 bars at KNZ).

| **Table S1.** ANPP-PPT regression results for each site (Figure 1). | | | | | | |  |
| --- | --- | --- | --- | --- | --- | --- | --- |
|  |  |  |  |  |  |  |  |
| Model: ANPP ~ Annual Precipitation | | | | | | |  |
|  |  | | Estimate | SE | t value | P-value | Model Adj. R^2^ |
| ALL | Intercept | | -36.56 | 23.62 | -1.548 | 0.126 | 0.724 |
|  | **Precipitation** | | 0.602 | 0.044 | 13.683 | **<2e-16** |  |
| KNZ | Intercept | | 93.44 | 95.01 | 0.983 | 0.340 | 0.447 |
|  | **Precipitation** | | 0.475 | 0.124 | 3.841 | **0.001** |  |
| HYS | Intercept | | 59.05 | 75.67 | 0.78 | 0.447 | 0.447 |
|  | **Precipitation** | | 0.473 | 0.123 | 3.838 | **0.001** |  |
| HPG | **Intercept** | | 112.31 | 26.5 | 4.238 | **0.001** | -0.062 |
|  | Precipitation | | -0.006 | 0.082 | -0.072 | 0.944 |  |
| SGS | Intercept | | 48.514 | 27.93 | 1.737 | 0.102 | 0.166 |
|  | Precipitation | | 0.206 | 0.099 | 2.089 | *0.053* |  |
|  |  |  |  |  |  |  |  |

| **Table S2.** Ambient yearly precipitation and 30-year mean annual precipitation (MAP) for each site (mm). Annual precipitation is listed first, followed by growing season precipitation in parentheses. White background indicates values for natural drought/recovery year (2012-2013) and 30-year MAP, blue background for the experimental drought period (2014-2017), and gray background for the experimental recovery period (2018-2021). Within each period (natural, experimental drought, and experimental recovery), the driest annual and growing season precipitation years are bolded and the wettest italicized. | | | | | | | | |  |
| --- | --- | --- | --- | --- | --- | --- | --- | --- | --- |
|  |  |  |  |  |  |  |  |  |  |
|  |  |  |  |  |  |  |  |  |  |
|  |  |  |  |  |  |  |  |  |  |
| **Year** | **KNZ** | | **HYS** | | **HPG** | | **SGS** | |  |
| 2012 | **568.90** | **(402.80)** | **366.00** | **(249.90)** | **259.00** | **(173.60)** | **217.50** | **(137.50)** |  |
| 2013 | *783.40* | *(574.80)* | *548.00* | *(393.70)* | *462.90* | *(333.80)* | *357.80* | *(274.00)* |  |
| 2014 | **635.57** | (476.04) | **458.68** | (390.10) | 333.00 | (223.02) | 293.24 | (220.36) |  |
| 2015 | *1002.50* | (725.80) | 513.34 | **(375.16)** | *355.09* | *(282.96)* | *368.55* | *(289.05)* |  |
| 2016 | 945.14 | *(763.50)* | *739.94* | *(652.79)* | **256.79** | **(211.33)** | **204.22** | **(152.91)** |  |
| 2017 | 720.20 | **(447.00)** | 570.49 | (411.49) | 281.43 | (215.90) | 288.54 | (221.23) |  |
| 2018 | 811.20 | (504.90) | 954.00 | (657.10) | 396.20 | *(298.90)* | 267.00 | (182.70) |  |
| 2019 | *1131.10* | *(865.70)* | 786.30 | (577.20) | *458.50* | (297.60) | 356.10 | (230.50) |  |
| 2020 | 807.90 | (564.50) | **562.30** | **(451.70)** | **255.40** | **(165.70)** | **249.30** | **(168.00)** |  |
| 2021 | **632.20** | **(420.50)** | 673.20 | (485.50) | 361.80 | (215.90) | *397.98* | *(286.28)* |  |
| MAP | 856.46 | (591.12) | 614.68 | (440.08) | 385.13 | (275.85) | 334.34 | (243.78) |  |

| **Table S3.** Yearly average daily mean temperatures and 30-year mean annual temperature (MAT) for each site (ºC). Annual temperatures are listed first, followed by growing season temperatures in parentheses. White background indicates values for natural drought/recovery year (2012-2013) and 30-year MAT, blue background for the experimental drought period (2014-2017), and gray background for the experimental recovery period (2018-2021). Within each period (natural, experimental drought, and experimental recovery), the hottest annual and growing season years are bolded and the coolest italicized. | | | | | | | | |  |
| --- | --- | --- | --- | --- | --- | --- | --- | --- | --- |
|  |  |  |  |  |  |  |  |  |  |
|  |  |  |  |  |  |  |  |  |  |
|  |  |  |  |  |  |  |  |  |  |
| **Year** | **KNZ** | | **HYS** | | **HPG** | | **SGS** | |  |
| 2012 | **14.37** | **(22.88)** | **14.24** | **(23.03)** | **9.02** | **(16.51)** | **9.80** | **(17.71)** |  |
| 2013 | *11.53* | *(20.17)* | *12.14* | *(20.88)* | *7.03* | *(14.61)* | *7.96* | *(15.80)* |  |
| 2014 | *11.85* | (20.89) | *12.01* | *(20.68)* | *7.30* | *(13.85)* | *7.97* | *(15.00)* |  |
| 2015 | 13.48 | (21.46) | **13.59** | **(21.54)** | 8.25 | (14.42) | 8.97 | (15.51) |  |
| 2016 | **14.28** | **(21.95)** | 13.55 | (21.08) | 8.32 | (14.55) | 8.97 | (15.56) |  |
| 2017 | 13.76 | *(20.88)* | 13.25 | (20.80) | **8.39** | **(14.89)** | **9.08** | **(15.92)** |  |
| 2018 | 12.70 | **(21.94)** | 12.10 | **(21.05)** | 7.78 | **(15.22)** | 8.14 | (15.93) |  |
| 2019 | *11.88* | (20.66) | *11.49* | (20.43) | *6.89* | *(14.24)* | *7.27* | *(15.20)* |  |
| 2020 | 12.69 | *(20.37)* | 12.51 | *(20.23)* | **8.16** | (15.04) | 8.74 | (16.00) |  |
| 2021 | **13.36** | (20.99) | **12.83** | (20.83) | 8.08 | (14.79) | **8.98** | **(16.25)** |  |
| MAT | 12.50 | (21.0) | 12.40 | (20.9) | 7.59 | (14.5) | 8.29 | (15.7) |  |

| **Table S4.** Yearly average mean daily VPD and 30-year VPD long-term averages (LTA) for each site (kPa). Annual values are listed first, followed by growing season in parentheses. White background indicates values for natural drought/recovery year (2012-2013) and 30-year LTA, blue background for the experimental drought period (2014-2017), and gray background for the experimental recovery period (2018-2021). Within each period (natural, experimental drought, and experimental recovery), the highest annual and growing season years are bolded and the lowest italicized. | | | | | | | | |  |
| --- | --- | --- | --- | --- | --- | --- | --- | --- | --- |
|  |  |  |  |  |  |  |  |  |  |
|  |  |  |  |  |  |  |  |  |  |
|  |  |  |  |  |  |  |  |  |  |
| **Year** | **KNZ** | | **HYS** | | **HPG** | | **SGS** | |  |
| 2012 | **1.10** | **(1.67)** | **1.32** | **(2.04)** | **1.00** | **(1.51)** | **1.12** | **(1.69)** |  |
| 2013 | *0.77* | *(1.11)* | *1.02* | *(1.55)* | *0.77* | *(1.18)* | *0.87* | *(1.32)* |  |
| 2014 | *0.83* | **(1.21)** | 1.03 | **(1.50)** | *0.75* | *(1.01)* | *0.85* | *(1.16)* |  |
| 2015 | 0.83 | *(1.09)* | **1.06** | (1.46) | 0.79 | (1.07) | 0.90 | (1.22) |  |
| 2016 | **0.86** | (1.15) | *1.03* | *(1.34)* | **0.85** | (1.17) | **0.98** | (1.34) |  |
| 2017 | 0.85 | (1.13) | 1.04 | (1.43) | 0.84 | **(1.20)** | 0.95 | **(1.35)** |  |
| 2018 | 0.84 | **(1.30)** | 0.92 | (1.35) | 0.80 | (1.18) | 0.92 | (1.37) |  |
| 2019 | *0.70* | *(1.01)* | *0.84* | *(1.24)* | *0.76* | *(1.09)* | *0.85* | *(1.26)* |  |
| 2020 | 0.78 | (1.05) | 0.94 | (1.29) | **0.95** | **(1.39)** | **1.06** | **(1.57)** |  |
| 2021 | **0.87** | (1.16) | **0.99** | **(1.35)** | 0.85 | (1.20) | 0.98 | (1.40) |  |
| VPD LTA | 0.81 | (1.16) | 1.00 | (1.45) | 0.79 | (1.14) | 0.89 | (1.30) |  |

| **Table S5.** Analyses and results for climate variables (Figure 2). | | | | | | |  |  |
| --- | --- | --- | --- | --- | --- | --- | --- | --- |
| ***One Sample T-test*** *comparing the percent of LTA precipitation during the 2012 natural drought compared to the average across the 2014-2017 experimental drought (n = 5 observations per site – one for each year). All experimental droughts showed a greater reduction from the LTA precipitation than the natural drought, though only HPG and SGS were significantly lower (or more severe).* | | | | | | | |  |
|  | % of LTA precipitation | |  |  |  | 95% CI | |  |
| Site | 2012 | 2014-2017 | t | df | p-value | Lower | Upper |  |
| KNZ | 66.395 | 53.952 | -1.845 | 3 | 0.1622 | 32.49 | 75.41 |  |
| HYS | 59.543 | 50.118 | -1.036 | 3 | 0.3765 | 21.15 | 79.08 |  |
| **HPG** | 67.249 | 38.241 | -6.517 | 3 | **0.0073** | 24.08 | 52.41 |  |
| **SGS** | 64.911 | 41.577 | -4.022 | 3 | **0.0276** | 23.11 | 60.03 |  |
| ***ANOVA*** *for growing season average daily mean temperatures across sites for the natural (2012) and experimental 2014-2017 drought periods (n = 20 observations, 5 per site for each year). Across all sites, mean daily temperatures for 2012 were significantly higher than during the experimental drought. Model adjusted R^2^ = 0.9851.* | | | | | | | |  |
| Model: Temperature ~ Site + Natural vs. Experimental | | | | | | | |  |
|  |  | Sum sq | | DF | F value | | P-value |  |
| **Intercept** |  | 1344.63 | | 1 | 8338.548 | | **<0.0001** |  |
| **Site** |  | 190.92 | | 3 | 394.654 | | **<0.0001** |  |
| **Natural vs. Experimental** | | 12.46 | | 1 | 77.289 | | **<0.0001** |  |
| Residuals |  | 2.42 | | 15 |  | |  |  |
| ***ANOVA*** *for growing season average daily mean VPD across sites for the natural (2012) and experimental (2014-2017) drought periods (n = 20 observations, 5 per site for each year). Across all sites, VPD was significantly higher in 2012 than during the experimental period. Model adjusted R^2^ = 0.9066.* | | | | | | | |  |
| Model: VPD ~ Site + Natural vs. Experimental | | | | | | | |  |
|  |  | Sum sq | | DF | F value | | P-value |  |
| **Intercept** |  | 6.7118 | | 1 | 1101.754 | | **<0.0001** |  |
| **Site** |  | 0.3819 | | 3 | 20.898 | | **<0.0001** |  |
| **Natural vs. Experimental** | | 0.7655 | | 1 | 125.658 | | **<0.0001** |  |
| Residuals |  | 0.0914 | | 15 |  | |  |  |
|  |  |  |  |  |  |  |  |  |

| **Table S6.** **Repeated measures mixed effects ANOVAs** for ANPP responses (g m^-2^) over the entire 10-year study (Figure 3a). Responses are split by period: the natural drought (2012) and recovery (2013) years, experimental drought (2014-2017) years, and experimental drought recovery (2018-2021) years. | | | | | | | | | | | | | | | | | | | | | | | | |  |
| --- | --- | --- | --- | --- | --- | --- | --- | --- | --- | --- | --- | --- | --- | --- | --- | --- | --- | --- | --- | --- | --- | --- | --- | --- | --- |
|  |  |  |  |  |  |  |  |  |  |  |  |  |  |  |  |  |  |  |  |  |  |  |  |  |  |
|  |  |  |  |  |  |  |  |  |  |  |  |  |  |  |  |  |  |  |  |  |  |  |  |  |  |
|  |  |  |  |  |  |  |  |  |  |  |  |  |  |  |  |  |  |  |  |  |  |  |  |  |  |
| ANPP (g m^-2^) responses during the 2012 natural drought vs. 2013 natural recovery year (n = 215 observations across all sites and both years). In 2012, the EDGE plots were not completed yet at KNZ, so measures come from n = 5 nearby plots. Since these plots are nearby, we include KNZ in the repeated measures ANOVA but results do not change whether KNZ is excluded or not. See Table S7 for responses of each year compared to LTAs. Model adjusted R^2^ = 0.8984. | | | | | | | | | | | | | | | | | | | | | | | | |  |
|  |  |  |  |  |  |  |  |  |  |  |  |  |  |  |  |  |  |  |  |  |  |  |  |  |  |
| Model: log(ANPP) ~ Site * Year + (1\|Plot) | | | | | | | | | | | | | | | | | | | | | | | | |  |
| \|  \| Sum sq \| Mean sq \| NumDF \| DenDF \| F value \| P-value \| \| --- \| --- \| --- \| --- \| --- \| --- \| --- \| \| **Site** \| 72.819 \| 24.2732 \| 3 \| 116.86 \| 290.55 \| **<0.0001** \| \| **Year** \| 16.243 \| 16.2433 \| 1 \| 140.09 \| 194.549 \| **<0.0001** \| \| **Year*Site** \| 6.888 \| 2.2961 \| 3 \| 116.53 \| 27.485 \| **<0.0001** \| \|  \|  \|  \|  \|  \|  \|  \| \| contrast \| \| estimate \| SE \| df \| t ratio \| P-value \| \| **KNZ 2012-2013** \| \| -0.666 \| 0.141 \| 182.9 \| -4.727 \| **<0.0001** \| \| **HYS 2012-2013** \| \| -0.729 \| 0.0774 \| 99.5 \| -9.429 \| **<0.0001** \| \| **HPG 2012-2013** \| \| -0.166 \| 0.0753 \| 93.7 \| -2.2025 \| **0.0299** \| \| **SGS 2012-2013** \| \| -1.128 \| 0.0753 \| 93.7 \| -14.99 \| **<0.0001** \| | | | | | | | | | | | | | | | | | | | | | | | | |  |
|  | | |  | | | |  | | | | |  | | | | |  | | | |  | | |  |  |
| *ANPP (g m^-2^) responses during the experimental drought period. Model adjusted R^2^ = 0.8478.* | | | | | | | | | | | | | | | | | | | | | | | | |  |
| Model: log(ANPP) ~ Site*Year*Treatment + (1\|Plot) | | | | | | | | | | | | | | | | | | | | | | | | |  |
|  | | | Sum sq | | | Mean sq | | | | NumDF | | | | | DenDF | | | | | F value | | | | P-value |  |
| **Site** | | | 77.62 | | | 25.8735 | | | | 3 | | | | | 112.88 | | | | | 384.7125 | | | | **<0.0001** |  |
| **Year** | | | 11.035 | | | 3.6784 | | | | 3 | | | | | 330.77 | | | | | 54.694 | | | | **<0.0001** |  |
| **Treatment** | | | 6.661 | | | 6.6609 | | | | 1 | | | | | 112.99 | | | | | 99.0411 | | | | **<0.0001** |  |
| **Site*Year** | | | 13.008 | | | 1.4454 | | | | 9 | | | | | 330.56 | | | | | 21.4912 | | | | **<0.0001** |  |
| **Site*Treatment** | | | 1.833 | | | 0.611 | | | | 3 | | | | | 112.88 | | | | | 9.0856 | | | | **<0.0001** |  |
| **Year*Treatment** | | | 0.802 | | | 0.2673 | | | | 3 | | | | | 330.77 | | | | | 3.9742 | | | | **0.0084** |  |
| **Site*Year*Treatment** | | | 1.526 | | | 0.1695 | | | | 9 | | | | | 330.56 | | | | | 2.521 | | | | **0.0083** |  |
|  | | |  | | | |  | | | | |  | | | | |  | | | |  | | |  |  |
| contrast | | | | estimate | | | | | SE | | | | | df | | | | t ratio | | | | P-value | | |  |
| **KNZ** | 2014 control-drought | | | 0.18018 | | | | | 0.108 | | | | | 418 | | | | 1.675 | | | | 0.0948 | | |  |
|  | **2015 control-drought** | | | 0.61402 | | | | | 0.108 | | | | | 418 | | | | 5.707 | | | | **<.0001** | | |  |
|  | **2016 control-drought** | | | 0.54976 | | | | | 0.108 | | | | | 418 | | | | 5.11 | | | | **<.0001** | | |  |
|  | **2017 control-drought** | | | 0.52791 | | | | | 0.108 | | | | | 418 | | | | 4.907 | | | | **<.0001** | | |  |
| **HYS** | **2014 control-drought** | | | 0.33398 | | | | | 0.116 | | | | | 426 | | | | 2.88 | | | | **0.0042** | | |  |
|  | **2015 control-drought** | | | 0.64834 | | | | | 0.111 | | | | | 421 | | | | 5.819 | | | | **<.0001** | | |  |
|  | **2016 control-drought** | | | 0.45547 | | | | | 0.112 | | | | | 423 | | | | 4.057 | | | | **0.0001** | | |  |
|  | **2017 control-drought** | | | 0.34606 | | | | | 0.111 | | | | | 421 | | | | 3.106 | | | | **0.002** | | |  |
|  |  | | |  | | | | |  | | | | |  | | | |  | | | |  | | |  |
| **HPG** | 2014 control-drought | | | 0.136 | | | | | 0.108 | | | | | 418 | | | | 1.278 | | | | 0.2069 | | |  |
|  | 2015 control-drought | | | 0.01743 | | | | | 0.108 | | | | | 418 | | | | 0.162 | | | | 0.8714 | | |  |
|  | 2016 control-drought | | | -0.0567 | | | | | 0.111 | | | | | 422 | | | | -0.531 | | | | 0.5958 | | |  |
|  | 2017 control-drought | | | 0.10124 | | | | | 0.108 | | | | | 418 | | | | 0.941 | | | | 0.3473 | | |  |
| **SGS** | **2014 control-drought** | | | 0.27194 | | | | | 0.109 | | | | | 419 | | | | 2.506 | | | | **0.0126** | | |  |
|  | **2015 control-drought** | | | 0.4747 | | | | | 0.108 | | | | | 418 | | | | 4.412 | | | | **<.0001** | | |  |
|  | 2016 control-drought | | | 0.00472 | | | | | 0.109 | | | | | 419 | | | | 0.044 | | | | 0.9653 | | |  |
|  | **2017 control-drought** | | | 0.49754 | | | | | 0.109 | | | | | 419 | | | | 4.586 | | | | **<.0001** | | |  |
|  | | |  | | | |  | | | | |  | | | | |  | | | |  | | |  |  |
| *ANPP (g m^-2^) recovery following the experimental drought period (2018-2021). Model adjusted R^2^ = 8772.* | | | | | | | | | | | | | | | | | | | | | | | | |  |
| Model: log(ANPP) ~ Site*Year*Treatment + (1\|Plot) | | | | | | | | | | | | | | | | | | | | | | | | |  |
|  | | | Sum sq | | | Mean sq | | | | | NumDF | | | | | DenDF | | | F value | | | | | P-value |  |
| **Site** | | | 123.956 | | | 41.319 | | | | | 3 | | | | | 112.07 | | | 452.1119 | | | | | **<0.0001** |  |
| **Year** | | | 14.952 | | | 4.984 | | | | | 3 | | | | | 331.24 | | | 54.5346 | | | | | **<0.0001** |  |
| Treatment | | | 0.006 | | | 0.006 | | | | | 1 | | | | | 112.08 | | | 0.0695 | | | | | 0.79257 |  |
| **Site*Year** | | | 20.8111 | | | 2.312 | | | | | 9 | | | | | 331.2 | | | 25.3014 | | | | | **<0.0001** |  |
| **Site*Treatment** | | | 0.974 | | | 0.325 | | | | | 3 | | | | | 112.07 | | | 3.5519 | | | | | **0.01675** |  |
| Year*Treatment | | | 0.607 | | | 0.202 | | | | | 3 | | | | | 331.24 | | | 2.2122 | | | | | 0.08653 |  |
| **Site*Year*Treatment** | | | 1.852 | | | 0.206 | | | | | 9 | | | | | 331.2 | | | 2.2518 | | | | | **0.01869** |  |
|  | | |  | | | |  | | | | |  | | | | |  | | | |  | | |  |  |
| contrast | | | | | estimate | | | SE | | | | | df | | | | | t ratio | | | | | P-value | |  |
| **KNZ** | | **2018 control-drought** | | | 0.4293 | | | 0.13 | | | | | 399 | | | | | 3.304 | | | | | **0.001** | |  |
|  |  | 2019 control-drought | | | 0.203 | | | 0.134 | | | | | 407 | | | | | 1.511 | | | | | 0.1316 | |  |
|  |  | 2020 control-drought | | | -0.0297 | | | 0.134 | | | | | 407 | | | | | -0.221 | | | | | 0.8254 | |  |
|  |  | 2021 control-drought | | | -0.0125 | | | 0.13 | | | | | 399 | | | | | -0.096 | | | | | 0.9233 | |  |
| **HYS** | | **2018 control-drought** | | | 0.2591 | | | 0.131 | | | | | 401 | | | | | 1.977 | | | | | **0.0487** | |  |
|  |  | 2019 control-drought | | | -0.1607 | | | 0.13 | | | | | 399 | | | | | -1.237 | | | | | 0.2169 | |  |
|  |  | 2020 control-drought | | | -0.056 | | | 0.131 | | | | | 401 | | | | | -0.427 | | | | | 0.6694 | |  |
|  |  | **2021 control-drought** | | | 0.3309 | | | 130 | | | | | 401 | | | | | 2.525 | | | | | **0.012** | |  |
| **HPG** | | 2018 control-drought | | | -0.0823 | | | 0.13 | | | | | 399 | | | | | -0.634 | | | | | 0.5268 | |  |
|  |  | 2019 control-drought | | | -0.0782 | | | 0.13 | | | | | 399 | | | | | -0.602 | | | | | 0.5474 | |  |
|  |  | **2020 control-drought** | | | -0.331 | | | 0.131 | | | | | 401 | | | | | -2.527 | | | | | **0.0119** | |  |
|  |  | 2021 control-drought | | | -0.1671 | | | 0.134 | | | | | 407 | | | | | -1.243 | | | | | 0.2144 | |  |
| **SGS** | | 2018 control-drought | | | -0.2406 | | | 0.13 | | | | | 399 | | | | | -1.851 | | | | | 0.0648 | |  |
|  |  | 2019 control-drought | | | -0.096 | | | 0.13 | | | | | 399 | | | | | -0.739 | | | | | 0.4604 | |  |
|  |  | 2020 control-drought | | | -0.0555 | | | 0.13 | | | | | 399 | | | | | -0.427 | | | | | 0.6696 | |  |
|  |  | 2021 control-drought | | | -0.0849 | | | 0.13 | | | | | 399 | | | | | -0.654 | | | | | 0.5137 | |  |

| **Table S7.** **Results of repeated measures ANOVA** for natural drought ANPP (g m^-2^) responses in 2012 (Figure 3b) and recovery in 2013 (Figure 5) compared to all other years. | | | | | | | |  |
| --- | --- | --- | --- | --- | --- | --- | --- | --- |
|  |  |  |  |  |  |  |  |  |
|  |  |  |  |  |  |  |  |  |
| ***ANOVA*** *of ANPP responses to the 2012 natural drought compared to 2013-2021 ambient ANPP (n = approx. 560 ambient plots across all sites and 10-yrs). All sites showed significant reductions in ANPP during the 2012 natural* drought*. Model adjusted R^2^ = 0.7989.* | | | | | | | |  |
| \| Model: log(ANPP) ~ Site * Period + (1\|Plot/Period) \| \| \| \| \| \| \| \| --- \| --- \| --- \| --- \| --- \| --- \| --- \| \|  \| Sum sq \| Mean sq \| NumDF \| DenDF \| F value \| P-value \| \| **Site** \| 106.284 \| 35.428 \| 3 \| 203.09 \| 207.412 \| **<0.0001** \| \| **Period** \| 32.707 \| 32.707 \| 1 \| 307.29 \| 191.609 \| **<0.0001** \| \| **Site * Period** \| 6.145 \| 2.048 \| 3 \| 203.09 \| 11.992 \| **<0.0001** \| \|  \|  \|  \|  \|  \|  \|  \| \| contrast \| \| estimate \| SE \| df \| t ratio \| P-value \| \| **KNZ 2012 vs. 9-yrs** \| \| -0.666 \| 0.1910 \| 455 \| -3.487 \| **0.0005** \| \| **HYS 2012 vs. 9-yrs** \| \| -1.023 \| 0.0873 \| 151 \| -11.718 \| **<0.0001** \| \| **HPG 2012 vs. 9-yrs** \| \| -0.493 \| 0.0857 \| 148 \| -5.757 \| **<0.0001** \| \| **SGS 2012 vs. 9-yrs** \| \| -1.173 \| 0.0856 \| 147 \| -13.710 \| **<0.0001** \| | | | | | | | |  |
|  |  |  |  |  |  |  |  |  |
| ***ANOVA*** *of ANPP responses during the 2013 recovery year from natural drought compared to 2012-2021 ambient ANPP (not including 2013; n = approx. 560 ambient plots across all sites and 10-yrs). KNZ and HYS showed no significant difference in ANPP in 2013 compared to their LTAs, but significant differences or potential legacies were evident at SGS and HPG. Model adjusted R^2^ = 0.6723.* | | | | | | | |  |
| \| Model: log(ANPP) ~ Site * Period + (1\|Plot/Period) \| \| \| \| \| \| \| \| --- \| --- \| --- \| --- \| --- \| --- \| --- \| \|  \| Sum sq \| Mean sq \| NumDF \| DenDF \| F value \| P-value \| \| **Site** \| 166.451 \| 55.484 \| 3 \| 128.70 \| 209.7271 \| **<0.0001** \| \| Period \| 0.200 \| 0.200 \| 1 \| 128.92 \| 0.7546 \| 0.3866 \| \| **Site * Period** \| 3.391 \| 1.130 \| 3 \| 182.70 \| 4.2728 \| **0.0065** \| \|  \|  \|  \|  \|  \|  \|  \| \| contrast \| \| estimate \| SE \| df \| t ratio \| P-value \| \| KNZ 2013 vs. 9-yrs \| \| 0.0572 \| 0.125 \| 118 \| 0.456 \| 0.6490 \| \| HYS 2013 vs. 9-yrs \| \| 0.0035 \| 0.124 \| 135 \| 0.028 \| 0.9778 \| \| HPG 2013 vs. 9-yrs \| \| -0.2251 \| 0.120 \| 131 \| -1.875 \| 0.0631 \| \| **SGS 2013 vs. 9-yrs** \| \| 0.3769 \| 0.120 \| 130 \| 3.143 \| **0.0021** \| | | | | | | | |  |
|  | | | | | | | |  |
|  |  |  |  |  |  |  |  |  |

| **Table S8. Results of repeated measures mixed effects ANOVAs** to compare ANPP responses (g m^-2^ mm^-1^) during natural (2012) and experimental (2014-2017) drought periods. | | | | | | | | | | | | | | | |  |  |  |  |  |  |
| --- | --- | --- | --- | --- | --- | --- | --- | --- | --- | --- | --- | --- | --- | --- | --- | --- | --- | --- | --- | --- | --- |
|  |  |  |  |  |  |  |  |  |  |  |  |  |  |  |  |  | | |  |  |  |
|  |  | | |  | | |  | | |  | | | | |  |  | | |  | | |
| *ANPP sensitivity response (g m^-2^ mm^-1^) in the first year of natural (2012) and experimental (2014) droughts. The Site*Year interaction effect was not significant, so the model was run again dropping that variable and with a cube root transformation of the response variable to meet model assumptions. Across all sites, the responses to natural and experimental drought were significantly different. Model adjusted R^2^ = 0.5175.* | | | | | | | | | | | | | | | |  | | |  |  |  |
| Model: ANPP^1/3^ ~ Site + Natural vs. Experimental + (1\|Block) | | | | | | | | | | | | | | | |  | | |  |  |  |
|  | Sum sq | Mean sq | | | | NumDF | | | DenDF | | F value | | P-value | | |  | | |  |  |  |
| **Site** | 0.4744 | 0.1581 | | | | 3 | | | 32.855 | | 7.7014 | | **0.0005** | | |  | | |  |  |  |
| **Natural vs. Experimental** | 1.0089 | 1.0089 | | | | 1 | | | 34.945 | | 49.1447 | | **<0.0001** | | |  | | |  |  |  |
|  | | | | | | | | | | | | | | | |  | | |  |  |  |
| *ANPP sensitivity response (g m^-2^ mm^-1^) to the natural drought (2012) compared to the average across experimental drought years (2014-2017). The Site*Year interaction effect was not significant, so the model was run again dropping that variable and with a log transformation of the response variable to meet model assumptions. Across all sites, the responses to natural and experimental drought were significantly different.* | | | | | | | | | | | | | | | |  | | |  |  |  |
| Model: ANPP ~ Site + Natural vs. Experimental + (1\|Block/Natural vs. Experimental) | | | | | | | | | | | | | | | | |  | | |  |  |
|  | Sum sq | | Mean sq | | NumDF | | | DenDF | | | | F value | | P-value | | | |  | | |  |
| **Site** | 6.6774 | | 2.2258 | | 3 | | | 35.935 | | | | 23.435 | | **<0.0001** | | | |  | | |  |
| **Natural vs. Experimental** | 6.4753 | | 6.4753 | | 1 | | | 64.704 | | | | 68.200 | | **<0.0001** | | | |  | | |  |
|  | | | | | | | | | | | | | | | |  | | |  |  |  |
|  |  |  |  |  |  |  |  |  |  |  |  |  |  |  |  |  | | |  |  |  |
|  |  |  |  |  |  |  |  |  |  |  |  |  |  |  |  |  | | |  |  |  |
|  |  |  |  |  |  |  |  |  |  |  |  |  |  |  |  |  | | |  |  |  |
|  |  |  |  |  |  |  |  |  |  |  |  |  |  |  |  |  | | |  |  |  |

| **Table S9. Simple linear regression ANOVAs** for ANPP responses (g m^-2^) during the first recovery year following experimental drought (2018) and averaged across the entire experimental drought recovery period (2018-2021; Figure 5). | | | | | | | | |
| --- | --- | --- | --- | --- | --- | --- | --- | --- |
|  |  |  |  |  |  |  |  |  |
|  |  |  |  |  |  |  |  |  |
|  |  |  |  |  |  |  |  |  |
| *ANOVA of ANPP responses during the 2018 recovery year from experimental drought in ambient and experimentally/simulated droughted plots. Significant differences of pairwise comparisons indicate legacy effects of the experimental drought at those sites.* | | | | | | | | |
| Model: log(ANPP) ~ Site * Treatment | | | | | | | | |
|  | Sum sq | | df | F value | | | | P-value |
| **Intercept** | 199.168 | | 1 | 2315.428 | | | | **<0.0001** |
| **Site** | 31.626 | | 3 | 122.555 | | | | **<0.0001** |
| Treatment | 0.045 | | 1 | 0.5253 | | | | 0.4701 |
| **Site*Treatment** | 1.901 | | 3 | 7.3685 | | | | **0.0002** |
| Residuals | 9.548 | | 111 |  | | | |  |
|  | |  |  | |  |  |  | |
| contrast | | estimate | SE | | df | t ratio | P-value | |
| **KNZ control vs. drought** | | 0.4293 | 0.114 | | 111 | 3.780 | **0.0003** | |
| **HYS control vs. drought** | | 0.2679 | 0.115 | | 111 | 2.338 | **0.0212** | |
| HPG control vs. drought | | -0.0823 | 0.114 | | 111 | -0.725 | 0.4701 | |
| **SGS control vs. drought** | | -0.2406 | 0.114 | | 111 | -2.118 | **0.0364** | |
|  | | | | | | | | |

*Repeated measures ANOVA of ANPP responses over the entire 2019-2021 recovery period following experimental drought in ambient and experimentally/simulated drought plots.*

| Model: log(ANPP) ~ Site * Treatment + (1\|Plot) | | | | | | |
| --- | --- | --- | --- | --- | --- | --- |
|  | Sum sq | Mean sq | NumDF | DenDF | F value | P-value |
| **Site** | 236.633 | 78.878 | 3 | 112.01 | 389.336 | **<0.0001** |
| Treatment | 0.024 | 0.024 | 1 | 112.04 | 0.1172 | 0.7327 |
| **Site * Treatment** | 1.864 | 0.621 | 3 | 112.01 | 3.0674 | **0.0309** |
|  |  |  |  |  |  |  |
| contrast | | estimate | SE | df | t ratio | P-value |
| KNZ control vs. drought | | 0.1366 | 0.0887 | 115 | 1.540 | 0.1264 |
| HYS control vs. drought | | 0.0962 | 0.0877 | 111 | 1.097 | 0.2752 |
| HPG control vs. drought | | -0.1737 | 0.0881 | 113 | -1.972 | 0.0511 |
| SGS control vs. drought | | -0.1193 | 0.0872 | 109 | -1.368 | 0.1740 |
